# Tris(2,2′-bipyridine-κ^2^ *N*,*N*′)cobalt(III) bis­[bis­(pyridine-2,6-dicarboxyl­ato-κ^3^ *O* ^2^,*N*,*O* ^6^)cobaltate(III)] perchlorate dimethyl­formamide hemisolvate 1.3-hydrate

**DOI:** 10.1107/S1600536812037208

**Published:** 2012-09-01

**Authors:** Irina A. Golenya, Alexander N. Boyko, Natalia V. Kotova, Matti Haukka, Turganbay S. Iskenderov

**Affiliations:** a Department of Chemistry, Kiev National Taras Shevchenko University, Volodymyrska Street 64, 01601 Kiev, Ukraine; b Department of Chemistry, University of Joensuu, PO Box 111, FI-80101 Joensuu, Finland

## Abstract

In the title compound, [Co(C_10_H_8_N_2_)_3_][Co(C_7_H_3_NO_4_)_2_]_2_(ClO_4_)·0.5C_3_H_7_NO·1.3H_2_O, the Co^III^ atom in the complex cation is pseudoocta­hedrally coordinated by six N atoms of three chelating bipyridine ligands. The Co^III^ atom in the complex anion is coordinated by two pyridine N atoms and four carboxyl­ate O atoms of two doubly deprotonated pyridine-2,6-dicarboxyl­ate ligands in a distorted octa­hedral geometry. One dimethyl­formamide solvent mol­ecule and two water mol­ecules are half-occupied and one water mol­ecule is 0.3-occupied. O—H⋯O hydrogen bonds link the water mol­ecules, the perchlorate anions and the complex anions. π–π inter­actions between the pyridine rings of the complex anions are also observed [centroid–centroid distance = 3.804 (3) Å].

## Related literature

For properties of polynuclear complexes, see: Fritsky *et al.* (2001 ▶, 2004 ▶); Krämer & Fritsky (2000 ▶); Moroz *et al.* (2010 ▶); Thompson (2002 ▶). For the use of hydroxamic acids in the synthesis of oligonuclear complexes and coordination polymers, see: Golenya *et al.* (2012*a* ▶,*b* ▶); Gumienna-Kontecka *et al.* (2007 ▶); Mezei *et al.* (2007 ▶); Pavlishchuk *et al.* (2011 ▶); Strotmeyer *et al.* (2004 ▶). For hydrolytic decomposition of hydroxamate ligands on complex formation, see: Dobosz *et al.* (1998 ▶, 1999 ▶); Świątek-Kozłowska *et al.* (2000 ▶). For the preparation of the ligand, see: Świątek-Kozłowska *et al.* (2002 ▶). For related structures, see: Fritsky *et al.* (2003 ▶); Mokhir *et al.* (2002 ▶); Moroz *et al.* (2008 ▶); Penkova *et al.* (2009 ▶); Sachse *et al.* (2008 ▶); Wörl *et al.* (2005*a* ▶,*b* ▶).
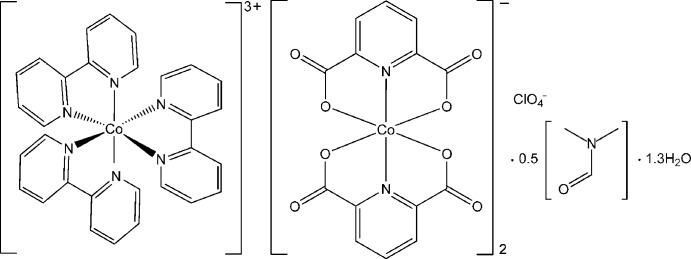


## Experimental

### 

#### Crystal data

[Co(C_10_H_8_N_2_)_3_][Co(C_7_H_3_NO_4_)_2_]_2_(ClO_4_)·0.5C_3_H_7_NO·1.3H_2_O
*M* *_r_* = 1465.18
Triclinic, 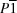

*a* = 14.1988 (4) Å
*b* = 14.7317 (6) Å
*c* = 16.6016 (8) Å
α = 113.286 (2)°
β = 107.128 (3)°
γ = 90.190 (3)°
*V* = 3019.8 (2) Å^3^
*Z* = 2
Mo *K*α radiation
μ = 0.95 mm^−1^
*T* = 100 K
0.32 × 0.18 × 0.08 mm

#### Data collection

Nonius KappaCCD diffractometer
Absorption correction: multi-scan (*DENZO*/*SCALEPACK*; Otwinowski & Minor, 1997 ▶) *T*_min_ = 0.753, *T*_max_ = 0.929
50715 measured reflections
13787 independent reflections
9788 reflections with *I* > 2σ(*I*)
*R*_int_ = 0.044

#### Refinement

*R*[*F* ^2^ > 2σ(*F* ^2^)] = 0.060
*wR*(*F* ^2^) = 0.181
*S* = 1.03
13787 reflections
871 parameters
1 restraint
H-atom parameters constrained
Δρ_max_ = 1.34 e Å^−3^
Δρ_min_ = −1.11 e Å^−3^

### 

Data collection: *COLLECT* (Nonius, 1998 ▶); cell refinement: *DENZO*/*SCALEPACK* (Otwinowski & Minor, 1997 ▶); data reduction: *DENZO*/*SCALEPACK*; program(s) used to solve structure: *SHELXS97* (Sheldrick, 2008 ▶); program(s) used to refine structure: *SHELXL97* (Sheldrick, 2008 ▶); molecular graphics: *DIAMOND* (Brandenburg, 1999 ▶); software used to prepare material for publication: *SHELXL97*.

## Supplementary Material

Crystal structure: contains datablock(s) I, global. DOI: 10.1107/S1600536812037208/hy2582sup1.cif

Structure factors: contains datablock(s) I. DOI: 10.1107/S1600536812037208/hy2582Isup2.hkl

Additional supplementary materials:  crystallographic information; 3D view; checkCIF report

## Figures and Tables

**Table 1:** Hydrogen-bond geometry (Å, °)

*D*—H⋯*A*	*D*—H	H⋯*A*	*D*⋯*A*	*D*—H⋯*A*
O14*A*—H14*E*⋯O12	0.94	2.11	3.017 (9)	164
O14*A*—H14*F*⋯O3*A* ^i^	0.94	1.97	2.861 (9)	156
O15—H115⋯O7*A* ^ii^	0.85	2.20	3.029 (13)	164
O15—H215⋯O2*B*	0.85	2.07	2.842 (13)	152

Symmetry codes: (i) 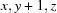; (ii) 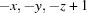.

**Fig. 1.**
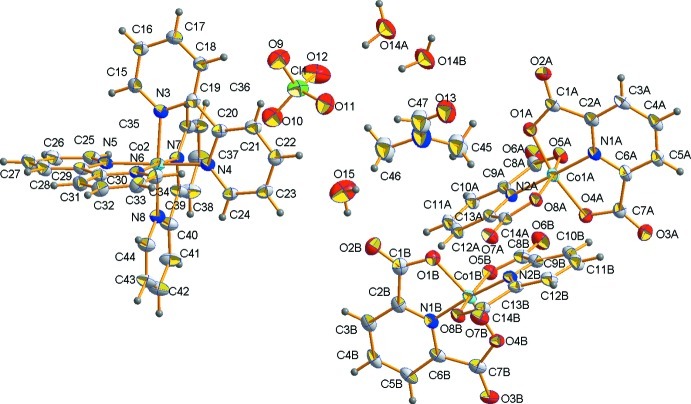
Molecular structure of the title compound, with displacement ellipsoids shown at the 50% probability level.

**Fig. 2.**
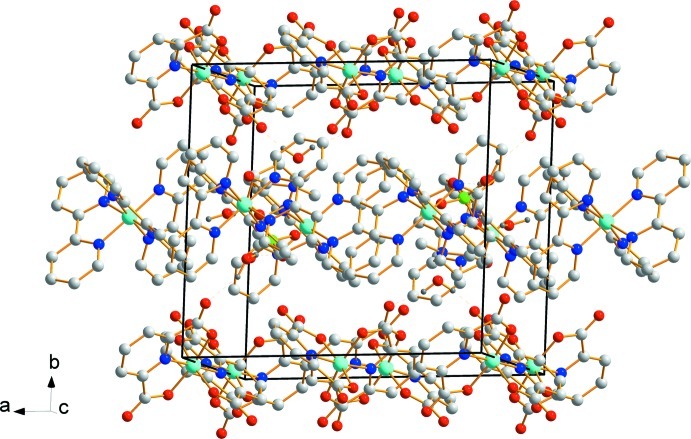
A packing diagram of the title compound. H atoms have been omitted for clarity.

## supplementary crystallographic information

### Comment

Polynuclear complexes and supramolecular assemblies containing both cationic and anionic modules are widely used in molecular magnetism, crystal engineering, bioinorganic modeling and catalysis (Fritsky *et al.*, 2001, 2004; Krämer & Fritsky, 2000; Moroz *et al.*, 2010; Thompson, 2002). Hydroxamic acids are extensively used in synthesis of discrete oligonuclear compounds (e.g. metallacrowns) (Golenya *et al.*, 2012*a*,b; Mezei *et al.*, 2007; Strotmeyer *et al.*, 2004) and coordination polymers (Gumienna-Kontecka *et al.*, 2007; Pavlishchuk *et al.*, 2011). However, the synthesis of such compounds in aqueous solution under alkaline conditions is sometimes complicated by hydrolytic decomposition of the hydroxamate function resulting in the formation of carboxylic groups (Dobosz *et al.*, 1998, 1999; Świątek-Kozłowska *et al.*, 2000). Herein we report the crystal and molecular structure of the title compound obtained in the course of our attempt to obtain a mixed ligand binuclear cobalt complex as a result of hydrolytic decomposition of pyridine-2,6-dihydroxamic acid.

The title compound is ionic and contains discrete tris(2,2'-bipyridine)cobalt(III) cations, bis(pyridine-2,6-dicarboxylato)cobalt(III) complex anions, perchlorate anions and solvent DMF and water molecules (Fig. 1). The Co^III^ atom in the complex cation is pseudooctahedrally coordinated by six N atoms of three chelating bipyridine ligands. The Co^III^ atoms in the complex anions are coordinated by two pyridine N atoms and four carboxylate O atoms of two doubly deprotonated pyridine-2,6-dicarboxylate ligands in a distorted octahedral geometry. The values of Co—O and Co—N bond distances in the complex anions are in a range of 1.834 (3)–1.838 (3) Å and 1.904 (3)–1.938 (3) Å, respectively. The Co—N distances range in the complex cation is 1.930 (3)–1.955 (3) Å. The observed values of Co—O and Co—N bond lengths are typical for realted cobalt(III) complexes (Fritsky *et al.*, 2003; Mokhir *et al.*, 2002; Świątek-Kozłowska *et al.*, 2000). This clearly indicates that the metal ions in both complex cation and anions are in trivalent state. The C—O bond lengths in the deprotonated carboxylate groups differ significantly [1.239 (2) and 1.292 (2) Å], which is typical for monodentately coordinated carboxylates (Wörl *et al.*, 2005*a*, *b*). The C—N and C—C bond lengths in the 2,2'-bipyridine ligands and in the pyridine-2,6-dicarboxylate ligands are normal for 2-substituted pyridine derivatives (Moroz *et al.*, 2008; Penkova *et al.*, 2009; Sachse *et al.*, 2008).

The crystal packing of the title compound is presented in Fig. 2. O—H···O hydrogen bonds link the water molecules, the perchlorate anions and the complex anions. π–π interactions between the pyridine rings of the complex anions are observed [centroid–centroid distance = 3.804 (3) Å].

### Experimental

Cobalt(II) perchlorate hexahydrate (0.037 g, 0.1 mmol) was dissolved in methanol (5 ml) and mixed with a solution of pyridine-2,6-dihydroxamic acid (0.039 g, 0.2 mmol) synthesized according to Świątek-Kozłowska *et al.* (2002) in dimethylformamide (5 ml), then to the obtained mixture a solution of sodium hydroxide (0.1 M, 4 ml) was added. In a separate vessel, cobalt(II) perchlorate hexahydrate (0.037 g, 0.1 mmol) was dissolved in methanol (5 ml) and mixed with a solution of 2,2'-bipyridine (0.312 g, 2 mmol) in methanol (5 ml). Then the two obtained solutions were mixed, and the obtained mixture was stirred at 60 C° for 30 min and filtered. Dark red crystals suitable for X-ray analysis were obtained by slow diffusion of diethyl ether into the resulting solution at room temperature within 72 h. They were filtered off and washed with diethyl ether (yield: 62%).

### Refinement

The DMF molecule was partially lost and therefore it was refined with occupancy of 0.5. The N13—C45 and N13—C46 distances in the DMF molecule were set to be equal. Also, C, N and O in DMF were refined with equal anisotropic displacement parameters. One of the water molecules was disordered over two sites with equal occupancies. Another water molecule was refined with occupancy factor of 0.3. The water H atoms were located from a difference Fourier map and constrained to ride on the parent atoms, with *U*_iso_(H) = 1.5*U*_eq_(O). Other H atoms were positioned geometrically and refined as riding atoms, with C—H = 0.95 (CH) and 0.98 (CH_3_) Å and *U*_iso_(H) = 1.2(1.5 for methyl)*U*_eq_(C). The highest residual electron density was found at 1.06 Å from H46C atom and the deepest hole at 0.69 Å from Cl1 atom.

### Crystal data

**Table:**

[Co(C_10_H_8_N_2_)_3_][Co(C_7_H_3_NO_4_)_2_]_2_(ClO_4_)·0.5C_3_H_7_NO·1.3H_2_O	*Z* = 2
*M**_r_* = 1465.18	*F*(000) = 1490
Triclinic, *P*1	*D*_x_ = 1.611 Mg m^−^^3^
Hall symbol: -P 1	Mo *K*α radiation, λ = 0.71073 Å
*a* = 14.1988 (4) Å	Cell parameters from 50715 reflections
*b* = 14.7317 (6) Å	θ = 2.3–27.5°
*c* = 16.6016 (8) Å	µ = 0.95 mm^−^^1^
α = 113.286 (2)°	*T* = 100 K
β = 107.128 (3)°	Plate, dark-red
γ = 90.190 (3)°	0.32 × 0.18 × 0.08 mm
*V* = 3019.8 (2) Å^3^	

### Data collection

**Table:**

Nonius KappaCCD diffractometer	13787 independent reflections
Radiation source: fine-focus sealed tube	9788 reflections with *I* > 2σ(*I*)
Horizontally mounted graphite crystal monochromator	*R*_int_ = 0.044
Detector resolution: 9 pixels mm^-1^	θ_max_ = 27.5°, θ_min_ = 2.3°
φ and ω scans	*h* = −18→18
Absorption correction: multi-scan (*DENZO*/*SCALEPACK*; Otwinowski & Minor, 1997)	*k* = −19→19
*T*_min_ = 0.753, *T*_max_ = 0.929	*l* = −21→21
50715 measured reflections	

### Refinement

**Table:**

Refinement on *F*^2^	Primary atom site location: structure-invariant direct methods
Least-squares matrix: full	Secondary atom site location: difference Fourier map
*R*[*F*^2^ > 2σ(*F*^2^)] = 0.060	Hydrogen site location: mixed
*wR*(*F*^2^) = 0.181	H-atom parameters constrained
*S* = 1.03	*w* = 1/[σ^2^(*F*_o_^2^) + (0.0882*P*)^2^ + 6.788*P*] where *P* = (*F*_o_^2^ + 2*F*_c_^2^)/3
13787 reflections	(Δ/σ)_max_ < 0.001
871 parameters	Δρ_max_ = 1.34 e Å^−^^3^
1 restraint	Δρ_min_ = −1.11 e Å^−^^3^

### Special details

**Table:**

Geometry. All esds (except the esd in the dihedral angle between two l.s. planes) are estimated using the full covariance matrix. The cell esds are taken into account individually in the estimation of esds in distances, angles and torsion angles; correlations between esds in cell parameters are only used when they are defined by crystal symmetry. An approximate (isotropic) treatment of cell esds is used for estimating esds involving l.s. planes.
Refinement. Refinement of F^2^ against ALL reflections. The weighted R-factor wR and goodness of fit S are based on F^2^, conventional R-factors R are based on F, with F set to zero for negative F^2^. The threshold expression of F^2^ > 2sigma(F^2^) is used only for calculating R-factors(gt) etc. and is not relevant to the choice of reflections for refinement. R-factors based on F^2^ are statistically about twice as large as those based on F, and R- factors based on ALL data will be even larger.

### Fractional atomic coordinates and isotropic or equivalent isotropic displacement parameters (Å^2^)

**Table:**

	*x*	*y*	*z*	*U*_iso_*/*U*_eq_	Occ. (<1)
Co1A	−0.00824 (4)	0.01102 (4)	0.78276 (4)	0.02594 (14)	
Co1B	0.47004 (4)	0.00821 (4)	0.71679 (4)	0.02581 (14)	
Co2	0.24444 (4)	0.50151 (4)	0.24937 (4)	0.02425 (14)	
Cl1	0.20408 (8)	0.57673 (9)	0.61001 (9)	0.0464 (3)	
O1A	−0.09182 (19)	0.1091 (2)	0.76943 (19)	0.0287 (6)	
O2A	−0.2162 (2)	0.1836 (2)	0.8138 (2)	0.0337 (6)	
O3A	0.0468 (2)	−0.1927 (2)	0.8807 (2)	0.0363 (7)	
O4A	0.0565 (2)	−0.0920 (2)	0.8104 (2)	0.0309 (6)	
O5A	0.0858 (2)	0.1126 (2)	0.88970 (19)	0.0321 (6)	
O6A	0.2252 (2)	0.2139 (3)	0.9317 (2)	0.0451 (8)	
O7A	−0.0809 (2)	−0.1502 (2)	0.5150 (2)	0.0361 (7)	
O8A	−0.08256 (19)	−0.0857 (2)	0.66155 (19)	0.0274 (6)	
O1B	0.4178 (2)	0.1164 (2)	0.6868 (2)	0.0311 (6)	
O2B	0.4444 (2)	0.2120 (2)	0.6165 (2)	0.0397 (7)	
O3B	0.6721 (2)	−0.1733 (2)	0.7022 (2)	0.0352 (7)	
O4B	0.54342 (19)	−0.0949 (2)	0.73398 (19)	0.0293 (6)	
O5B	0.5412 (2)	0.1005 (2)	0.84153 (19)	0.0310 (6)	
O6B	0.5385 (2)	0.1466 (2)	0.9873 (2)	0.0432 (8)	
O7B	0.2357 (2)	−0.1898 (2)	0.5513 (2)	0.0373 (7)	
O8B	0.37790 (19)	−0.0869 (2)	0.60488 (19)	0.0286 (6)	
O9	0.1282 (2)	0.6308 (3)	0.5794 (3)	0.0584 (10)	
O10	0.2684 (2)	0.5537 (3)	0.5536 (3)	0.0562 (10)	
O11	0.1580 (3)	0.4859 (3)	0.6027 (3)	0.0550 (9)	
O12	0.2630 (3)	0.6361 (3)	0.7060 (3)	0.0688 (12)	
O13	0.1629 (6)	0.3704 (7)	0.8102 (6)	0.0662 (13)	0.50
O14A	0.1748 (6)	0.6559 (7)	0.8558 (5)	0.0680 (16)	0.50
H14E	0.1935	0.6380	0.8024	0.102*	0.50
H14F	0.1499	0.7177	0.8730	0.102*	0.50
O14B	0.0973 (6)	0.4973 (7)	0.8239 (5)	0.0680 (16)	0.50
H14G	0.1070	0.4938	0.7717	0.102*	0.50
H14H	0.0384	0.5161	0.8356	0.102*	0.50
O15	0.2704 (8)	0.2910 (11)	0.5509 (11)	0.071 (4)	0.30
H115	0.2182	0.2563	0.5437	0.107*	0.30
H215	0.3167	0.2546	0.5502	0.107*	0.30
N1A	−0.0896 (2)	−0.0084 (2)	0.8440 (2)	0.0257 (7)	
N2A	0.0728 (2)	0.0334 (2)	0.7226 (2)	0.0248 (7)	
N1B	0.5602 (2)	0.0223 (2)	0.6615 (2)	0.0271 (7)	
N2B	0.3829 (2)	−0.0185 (2)	0.7699 (2)	0.0257 (7)	
N3	0.1579 (2)	0.5970 (2)	0.2940 (2)	0.0259 (7)	
N4	0.1906 (2)	0.4303 (2)	0.3052 (2)	0.0259 (7)	
N5	0.2875 (2)	0.5710 (2)	0.1848 (2)	0.0271 (7)	
N6	0.1394 (2)	0.4356 (2)	0.1312 (2)	0.0258 (7)	
N7	0.3557 (2)	0.5647 (2)	0.3636 (2)	0.0271 (7)	
N8	0.3362 (2)	0.4074 (2)	0.2137 (2)	0.0282 (7)	
N9	0.3161 (7)	0.4078 (8)	0.8183 (7)	0.0662 (13)	0.50
C1A	−0.1600 (3)	0.1209 (3)	0.8104 (3)	0.0276 (8)	
C2A	−0.1612 (3)	0.0488 (3)	0.8545 (3)	0.0279 (8)	
C3A	−0.2224 (3)	0.0372 (3)	0.9016 (3)	0.0308 (9)	
H3A	−0.2739	0.0773	0.9104	0.037*	
C4A	−0.2058 (3)	−0.0361 (3)	0.9361 (3)	0.0331 (9)	
H4A	−0.2472	−0.0462	0.9684	0.040*	
C5A	−0.1300 (3)	−0.0944 (3)	0.9241 (3)	0.0308 (9)	
H5A	−0.1189	−0.1439	0.9478	0.037*	
C6A	−0.0713 (3)	−0.0781 (3)	0.8769 (3)	0.0283 (8)	
C7A	0.0173 (3)	−0.1271 (3)	0.8555 (3)	0.0294 (9)	
C8A	0.1575 (3)	0.1501 (3)	0.8729 (3)	0.0325 (9)	
C9A	0.1505 (3)	0.1049 (3)	0.7724 (3)	0.0268 (8)	
C10A	0.2110 (3)	0.1266 (3)	0.7287 (3)	0.0310 (9)	
H10A	0.2667	0.1773	0.7627	0.037*	
C11A	0.1882 (3)	0.0725 (3)	0.6340 (3)	0.0326 (9)	
H11A	0.2285	0.0868	0.6027	0.039*	
C12A	0.1065 (3)	−0.0031 (3)	0.5836 (3)	0.0308 (9)	
H12A	0.0913	−0.0411	0.5188	0.037*	
C13A	0.0486 (3)	−0.0202 (3)	0.6318 (3)	0.0270 (8)	
C14A	−0.0448 (3)	−0.0928 (3)	0.5966 (3)	0.0273 (8)	
C1B	0.4648 (3)	0.1453 (3)	0.6423 (3)	0.0322 (9)	
C2B	0.5510 (3)	0.0889 (3)	0.6260 (3)	0.0296 (9)	
C3B	0.6147 (3)	0.0978 (3)	0.5797 (3)	0.0329 (9)	
H3B	0.6097	0.1460	0.5544	0.040*	
C4B	0.6863 (3)	0.0337 (3)	0.5716 (3)	0.0363 (10)	
H4B	0.7314	0.0385	0.5406	0.044*	
C5B	0.6934 (3)	−0.0372 (3)	0.6079 (3)	0.0343 (10)	
H5B	0.7416	−0.0818	0.6011	0.041*	
C6B	0.6272 (3)	−0.0406 (3)	0.6545 (3)	0.0283 (9)	
C7B	0.6166 (3)	−0.1097 (3)	0.6996 (3)	0.0304 (9)	
C8B	0.5037 (3)	0.0971 (3)	0.9037 (3)	0.0316 (9)	
C9B	0.4105 (3)	0.0214 (3)	0.8618 (3)	0.0287 (8)	
C10B	0.3597 (3)	−0.0137 (3)	0.9052 (3)	0.0328 (9)	
H10B	0.3790	0.0133	0.9709	0.039*	
C11B	0.2805 (3)	−0.0886 (3)	0.8515 (3)	0.0369 (10)	
H11B	0.2444	−0.1128	0.8805	0.044*	
C12B	0.2526 (3)	−0.1291 (3)	0.7549 (3)	0.0318 (9)	
H12B	0.1975	−0.1801	0.7176	0.038*	
C13B	0.3078 (3)	−0.0925 (3)	0.7155 (3)	0.0283 (8)	
C14B	0.3028 (3)	−0.1280 (3)	0.6151 (3)	0.0287 (8)	
C15	0.1501 (3)	0.6856 (3)	0.2884 (3)	0.0285 (8)	
H15	0.1879	0.7047	0.2576	0.034*	
C16	0.0887 (3)	0.7492 (3)	0.3261 (3)	0.0330 (9)	
H16	0.0846	0.8115	0.3216	0.040*	
C17	0.0329 (3)	0.7218 (3)	0.3709 (3)	0.0370 (10)	
H17	−0.0097	0.7649	0.3976	0.044*	
C18	0.0403 (3)	0.6306 (3)	0.3758 (3)	0.0353 (10)	
H18	0.0026	0.6100	0.4059	0.042*	
C19	0.1027 (3)	0.5701 (3)	0.3369 (3)	0.0285 (8)	
C20	0.1182 (3)	0.4729 (3)	0.3400 (3)	0.0283 (8)	
C21	0.0650 (3)	0.4264 (3)	0.3739 (3)	0.0354 (10)	
H21	0.0136	0.4567	0.3964	0.042*	
C22	0.0871 (3)	0.3358 (3)	0.3749 (3)	0.0379 (10)	
H22	0.0509	0.3029	0.3978	0.045*	
C23	0.1631 (3)	0.2931 (3)	0.3419 (3)	0.0333 (9)	
H23	0.1805	0.2314	0.3433	0.040*	
C24	0.2128 (3)	0.3419 (3)	0.3072 (3)	0.0277 (8)	
H24	0.2641	0.3123	0.2840	0.033*	
C25	0.3678 (3)	0.6408 (3)	0.2189 (3)	0.0334 (9)	
H25	0.4096	0.6606	0.2810	0.040*	
C26	0.3904 (3)	0.6844 (3)	0.1651 (3)	0.0366 (10)	
H26	0.4476	0.7328	0.1904	0.044*	
C27	0.3303 (3)	0.6575 (3)	0.0755 (3)	0.0388 (10)	
H27	0.3438	0.6883	0.0388	0.047*	
C28	0.2491 (3)	0.5839 (3)	0.0398 (3)	0.0350 (10)	
H28	0.2069	0.5629	−0.0224	0.042*	
C29	0.2304 (3)	0.5420 (3)	0.0951 (3)	0.0280 (8)	
C30	0.1471 (3)	0.4627 (3)	0.0636 (3)	0.0285 (8)	
C31	0.0823 (3)	0.4182 (3)	−0.0262 (3)	0.0322 (9)	
H31	0.0896	0.4378	−0.0725	0.039*	
C32	0.0066 (3)	0.3445 (3)	−0.0475 (3)	0.0336 (9)	
H32	−0.0380	0.3117	−0.1089	0.040*	
C33	−0.0031 (3)	0.3197 (3)	0.0210 (3)	0.0339 (9)	
H33	−0.0556	0.2706	0.0079	0.041*	
C34	0.0638 (3)	0.3664 (3)	0.1094 (3)	0.0305 (9)	
H34	0.0559	0.3487	0.1565	0.037*	
C35	0.3568 (3)	0.6453 (3)	0.4396 (3)	0.0291 (9)	
H35	0.2974	0.6745	0.4415	0.035*	
C36	0.4426 (3)	0.6868 (3)	0.5152 (3)	0.0354 (10)	
H36	0.4418	0.7438	0.5683	0.042*	
C37	0.5292 (3)	0.6450 (3)	0.5129 (3)	0.0425 (11)	
H37	0.5891	0.6743	0.5633	0.051*	
C38	0.5275 (3)	0.5599 (3)	0.4363 (3)	0.0421 (11)	
H38	0.5858	0.5290	0.4339	0.051*	
C39	0.4392 (3)	0.5200 (3)	0.3627 (3)	0.0317 (9)	
C40	0.4282 (3)	0.4297 (3)	0.2788 (3)	0.0323 (9)	
C41	0.5023 (3)	0.3707 (3)	0.2644 (3)	0.0400 (11)	
H41	0.5650	0.3863	0.3116	0.048*	
C42	0.4849 (3)	0.2891 (3)	0.1813 (3)	0.0428 (11)	
H42	0.5352	0.2481	0.1702	0.051*	
C43	0.3930 (3)	0.2681 (3)	0.1145 (3)	0.0375 (10)	
H43	0.3799	0.2132	0.0560	0.045*	
C44	0.3199 (3)	0.3280 (3)	0.1334 (3)	0.0316 (9)	
H44	0.2562	0.3118	0.0876	0.038*	
C45	0.3650 (8)	0.3793 (10)	0.8971 (9)	0.0662 (13)	0.50
H45A	0.4374	0.3950	0.9157	0.099*	0.50
H45B	0.3473	0.3076	0.8771	0.099*	0.50
H45C	0.3422	0.4166	0.9498	0.099*	0.50
C46	0.3758 (8)	0.4354 (10)	0.7676 (9)	0.0662 (13)	0.50
H46A	0.4469	0.4442	0.8016	0.099*	0.50
H46B	0.3578	0.4978	0.7630	0.099*	0.50
H46C	0.3614	0.3821	0.7052	0.099*	0.50
C47	0.2156 (9)	0.3929 (10)	0.7803 (10)	0.0662 (13)	0.50
H47A	0.1881	0.4021	0.7250	0.079*	0.50

### Atomic displacement parameters (Å^2^)

**Table:**

	*U*^11^	*U*^22^	*U*^33^	*U*^12^	*U*^13^	*U*^23^
Co1A	0.0259 (3)	0.0289 (3)	0.0281 (3)	0.0028 (2)	0.0145 (2)	0.0127 (2)
Co1B	0.0234 (3)	0.0259 (3)	0.0276 (3)	−0.0002 (2)	0.0114 (2)	0.0083 (2)
Co2	0.0228 (3)	0.0198 (3)	0.0249 (3)	−0.00180 (19)	0.0081 (2)	0.0037 (2)
Cl1	0.0279 (5)	0.0419 (6)	0.0645 (8)	0.0021 (4)	0.0146 (5)	0.0173 (6)
O1A	0.0286 (14)	0.0292 (14)	0.0308 (15)	0.0046 (11)	0.0126 (12)	0.0126 (12)
O2A	0.0334 (15)	0.0297 (15)	0.0377 (17)	0.0073 (12)	0.0152 (13)	0.0111 (13)
O3A	0.0381 (16)	0.0347 (16)	0.0400 (17)	0.0039 (13)	0.0123 (14)	0.0195 (14)
O4A	0.0268 (14)	0.0343 (15)	0.0384 (16)	0.0052 (11)	0.0155 (12)	0.0183 (13)
O5A	0.0299 (14)	0.0394 (16)	0.0274 (15)	0.0016 (12)	0.0132 (12)	0.0114 (13)
O6A	0.0347 (16)	0.053 (2)	0.0334 (17)	−0.0078 (14)	0.0098 (14)	0.0050 (15)
O7A	0.0328 (15)	0.0391 (17)	0.0304 (16)	0.0011 (13)	0.0117 (13)	0.0074 (14)
O8A	0.0272 (13)	0.0289 (14)	0.0290 (15)	0.0006 (11)	0.0136 (11)	0.0117 (12)
O1B	0.0274 (14)	0.0293 (15)	0.0365 (16)	0.0001 (11)	0.0117 (12)	0.0127 (13)
O2B	0.0400 (17)	0.0337 (16)	0.0456 (19)	−0.0005 (13)	0.0092 (14)	0.0198 (15)
O3B	0.0283 (14)	0.0316 (15)	0.0372 (17)	0.0057 (12)	0.0101 (12)	0.0061 (13)
O4B	0.0266 (13)	0.0279 (14)	0.0339 (16)	0.0044 (11)	0.0121 (12)	0.0114 (12)
O5B	0.0282 (14)	0.0299 (15)	0.0312 (15)	−0.0005 (11)	0.0100 (12)	0.0088 (12)
O6B	0.0381 (17)	0.0498 (19)	0.0304 (17)	−0.0017 (14)	0.0083 (14)	0.0073 (15)
O7B	0.0318 (15)	0.0335 (16)	0.0364 (17)	−0.0034 (12)	0.0115 (13)	0.0040 (13)
O8B	0.0265 (13)	0.0303 (14)	0.0275 (14)	0.0000 (11)	0.0116 (11)	0.0084 (12)
O9	0.0290 (17)	0.064 (2)	0.093 (3)	0.0103 (16)	0.0218 (18)	0.042 (2)
O10	0.0348 (18)	0.065 (2)	0.080 (3)	0.0096 (16)	0.0272 (18)	0.035 (2)
O11	0.052 (2)	0.052 (2)	0.064 (2)	−0.0035 (16)	0.0189 (18)	0.0266 (19)
O12	0.039 (2)	0.062 (2)	0.071 (3)	0.0018 (17)	0.0099 (19)	0.000 (2)
O13	0.050 (2)	0.088 (3)	0.080 (3)	0.010 (2)	0.032 (2)	0.046 (3)
O14A	0.076 (4)	0.093 (4)	0.041 (3)	0.028 (3)	0.028 (3)	0.027 (3)
O14B	0.076 (4)	0.093 (4)	0.041 (3)	0.028 (3)	0.028 (3)	0.027 (3)
O15	0.026 (6)	0.077 (9)	0.098 (11)	0.006 (6)	0.002 (6)	0.037 (8)
N1A	0.0234 (15)	0.0284 (17)	0.0246 (17)	0.0010 (13)	0.0101 (13)	0.0088 (14)
N2A	0.0238 (15)	0.0275 (16)	0.0284 (17)	0.0039 (12)	0.0131 (13)	0.0136 (14)
N1B	0.0219 (15)	0.0288 (17)	0.0263 (17)	−0.0034 (13)	0.0081 (13)	0.0071 (14)
N2B	0.0227 (15)	0.0254 (16)	0.0286 (18)	0.0031 (12)	0.0107 (13)	0.0091 (14)
N3	0.0234 (15)	0.0254 (16)	0.0247 (17)	0.0000 (12)	0.0073 (13)	0.0064 (14)
N4	0.0252 (16)	0.0217 (16)	0.0253 (17)	−0.0014 (12)	0.0068 (13)	0.0054 (13)
N5	0.0259 (16)	0.0211 (15)	0.0303 (18)	−0.0003 (12)	0.0129 (14)	0.0040 (14)
N6	0.0243 (15)	0.0215 (15)	0.0270 (17)	0.0000 (12)	0.0079 (13)	0.0056 (13)
N7	0.0255 (16)	0.0221 (16)	0.0309 (18)	−0.0004 (12)	0.0098 (14)	0.0076 (14)
N8	0.0242 (16)	0.0243 (16)	0.0328 (18)	0.0002 (13)	0.0101 (14)	0.0080 (14)
N9	0.050 (2)	0.088 (3)	0.080 (3)	0.010 (2)	0.032 (2)	0.046 (3)
C1A	0.0258 (18)	0.028 (2)	0.0228 (19)	−0.0015 (16)	0.0090 (15)	0.0030 (16)
C2A	0.0269 (19)	0.027 (2)	0.0220 (19)	−0.0030 (15)	0.0066 (15)	0.0040 (16)
C3A	0.0246 (19)	0.034 (2)	0.024 (2)	−0.0025 (16)	0.0099 (16)	0.0014 (17)
C4A	0.033 (2)	0.037 (2)	0.026 (2)	−0.0082 (17)	0.0151 (17)	0.0062 (18)
C5A	0.035 (2)	0.029 (2)	0.025 (2)	−0.0068 (16)	0.0109 (17)	0.0073 (17)
C6A	0.030 (2)	0.030 (2)	0.0218 (19)	−0.0046 (16)	0.0088 (16)	0.0077 (16)
C7A	0.0276 (19)	0.032 (2)	0.030 (2)	0.0009 (16)	0.0103 (16)	0.0133 (18)
C8A	0.029 (2)	0.040 (2)	0.029 (2)	0.0052 (17)	0.0117 (17)	0.0134 (19)
C9A	0.0252 (18)	0.030 (2)	0.028 (2)	0.0056 (15)	0.0110 (16)	0.0121 (17)
C10A	0.0247 (19)	0.037 (2)	0.036 (2)	0.0017 (16)	0.0134 (17)	0.0168 (19)
C11A	0.0271 (19)	0.044 (2)	0.037 (2)	0.0054 (17)	0.0175 (18)	0.022 (2)
C12A	0.0270 (19)	0.040 (2)	0.030 (2)	0.0053 (17)	0.0141 (17)	0.0158 (18)
C13A	0.0261 (18)	0.030 (2)	0.030 (2)	0.0063 (15)	0.0135 (16)	0.0140 (17)
C14A	0.0244 (18)	0.030 (2)	0.033 (2)	0.0074 (15)	0.0129 (16)	0.0149 (18)
C1B	0.028 (2)	0.031 (2)	0.030 (2)	−0.0066 (16)	0.0038 (17)	0.0095 (18)
C2B	0.0283 (19)	0.029 (2)	0.024 (2)	−0.0081 (16)	0.0063 (16)	0.0052 (17)
C3B	0.034 (2)	0.033 (2)	0.025 (2)	−0.0083 (17)	0.0092 (17)	0.0056 (17)
C4B	0.031 (2)	0.047 (3)	0.026 (2)	−0.0098 (18)	0.0123 (17)	0.0087 (19)
C5B	0.0243 (19)	0.044 (2)	0.025 (2)	−0.0020 (17)	0.0104 (16)	0.0028 (18)
C6B	0.0220 (18)	0.030 (2)	0.0221 (19)	−0.0031 (15)	0.0063 (15)	0.0011 (16)
C7B	0.0228 (18)	0.030 (2)	0.028 (2)	−0.0023 (16)	0.0062 (16)	0.0026 (17)
C8B	0.0245 (19)	0.033 (2)	0.035 (2)	0.0041 (16)	0.0100 (17)	0.0108 (18)
C9B	0.0283 (19)	0.030 (2)	0.028 (2)	0.0069 (16)	0.0131 (16)	0.0090 (17)
C10B	0.030 (2)	0.041 (2)	0.030 (2)	0.0099 (17)	0.0152 (17)	0.0127 (19)
C11B	0.031 (2)	0.047 (3)	0.042 (3)	0.0077 (18)	0.0212 (19)	0.021 (2)
C12B	0.0231 (18)	0.031 (2)	0.040 (2)	0.0013 (15)	0.0142 (17)	0.0108 (19)
C13B	0.0263 (19)	0.0243 (19)	0.036 (2)	0.0058 (15)	0.0157 (17)	0.0100 (17)
C14B	0.0260 (19)	0.027 (2)	0.032 (2)	0.0039 (15)	0.0117 (17)	0.0090 (17)
C15	0.031 (2)	0.026 (2)	0.023 (2)	−0.0005 (15)	0.0053 (16)	0.0080 (16)
C16	0.040 (2)	0.027 (2)	0.031 (2)	0.0051 (17)	0.0116 (18)	0.0103 (18)
C17	0.041 (2)	0.035 (2)	0.040 (3)	0.0130 (18)	0.021 (2)	0.014 (2)
C18	0.037 (2)	0.032 (2)	0.045 (3)	0.0073 (17)	0.024 (2)	0.016 (2)
C19	0.0266 (19)	0.027 (2)	0.033 (2)	0.0022 (15)	0.0117 (16)	0.0117 (17)
C20	0.0241 (18)	0.0233 (19)	0.034 (2)	0.0011 (15)	0.0098 (16)	0.0079 (17)
C21	0.031 (2)	0.032 (2)	0.046 (3)	0.0052 (17)	0.0191 (19)	0.015 (2)
C22	0.037 (2)	0.031 (2)	0.054 (3)	0.0022 (17)	0.022 (2)	0.020 (2)
C23	0.035 (2)	0.0230 (19)	0.040 (2)	0.0033 (16)	0.0122 (18)	0.0112 (18)
C24	0.0257 (18)	0.0220 (19)	0.032 (2)	0.0013 (15)	0.0090 (16)	0.0080 (16)
C25	0.029 (2)	0.027 (2)	0.035 (2)	−0.0038 (16)	0.0120 (17)	0.0019 (17)
C26	0.037 (2)	0.028 (2)	0.043 (3)	−0.0040 (17)	0.024 (2)	0.0054 (19)
C27	0.043 (2)	0.035 (2)	0.041 (3)	−0.0008 (19)	0.024 (2)	0.011 (2)
C28	0.036 (2)	0.033 (2)	0.034 (2)	−0.0013 (17)	0.0156 (18)	0.0085 (19)
C29	0.0258 (19)	0.0240 (19)	0.030 (2)	0.0013 (15)	0.0142 (16)	0.0039 (16)
C30	0.0258 (19)	0.026 (2)	0.033 (2)	0.0041 (15)	0.0135 (16)	0.0093 (17)
C31	0.035 (2)	0.030 (2)	0.028 (2)	0.0009 (17)	0.0085 (17)	0.0091 (17)
C32	0.030 (2)	0.031 (2)	0.029 (2)	0.0005 (16)	0.0013 (17)	0.0080 (18)
C33	0.031 (2)	0.027 (2)	0.037 (2)	−0.0050 (16)	0.0051 (18)	0.0109 (18)
C34	0.029 (2)	0.0236 (19)	0.034 (2)	−0.0027 (15)	0.0079 (17)	0.0092 (17)
C35	0.0282 (19)	0.0224 (19)	0.030 (2)	−0.0018 (15)	0.0098 (16)	0.0040 (16)
C36	0.035 (2)	0.029 (2)	0.030 (2)	0.0023 (17)	0.0064 (18)	0.0022 (18)
C37	0.029 (2)	0.038 (2)	0.038 (3)	0.0001 (18)	−0.0004 (19)	0.001 (2)
C38	0.026 (2)	0.038 (2)	0.043 (3)	0.0054 (18)	0.0036 (19)	0.004 (2)
C39	0.0264 (19)	0.027 (2)	0.033 (2)	0.0014 (15)	0.0095 (17)	0.0032 (17)
C40	0.0252 (19)	0.030 (2)	0.032 (2)	0.0013 (16)	0.0091 (17)	0.0031 (18)
C41	0.026 (2)	0.036 (2)	0.042 (3)	0.0064 (17)	0.0061 (18)	0.004 (2)
C42	0.032 (2)	0.041 (3)	0.043 (3)	0.0088 (19)	0.015 (2)	0.003 (2)
C43	0.036 (2)	0.030 (2)	0.033 (2)	0.0026 (17)	0.0134 (19)	−0.0022 (18)
C44	0.028 (2)	0.027 (2)	0.030 (2)	−0.0013 (16)	0.0072 (17)	0.0036 (17)
C45	0.050 (2)	0.088 (3)	0.080 (3)	0.010 (2)	0.032 (2)	0.046 (3)
C46	0.050 (2)	0.088 (3)	0.080 (3)	0.010 (2)	0.032 (2)	0.046 (3)
C47	0.050 (2)	0.088 (3)	0.080 (3)	0.010 (2)	0.032 (2)	0.046 (3)

### Geometric parameters (Å, º)

**Table:**

Co1A—N2A	1.834 (3)	C11A—C12A	1.403 (6)
Co1A—N1A	1.836 (3)	C11A—H11A	0.9500
Co1A—O1A	1.912 (3)	C12A—C13A	1.388 (5)
Co1A—O5A	1.914 (3)	C12A—H12A	0.9500
Co1A—O8A	1.915 (3)	C13A—C14A	1.512 (5)
Co1A—O4A	1.916 (3)	C1B—C2B	1.511 (6)
Co1B—N1B	1.835 (3)	C2B—C3B	1.384 (6)
Co1B—N2B	1.838 (3)	C3B—C4B	1.390 (6)
Co1B—O8B	1.904 (3)	C3B—H3B	0.9500
Co1B—O4B	1.912 (3)	C4B—C5B	1.388 (7)
Co1B—O5B	1.921 (3)	C4B—H4B	0.9500
Co1B—O1B	1.938 (3)	C5B—C6B	1.395 (5)
Co2—N4	1.930 (3)	C5B—H5B	0.9500
Co2—N3	1.939 (3)	C6B—C7B	1.512 (6)
Co2—N6	1.943 (3)	C8B—C9B	1.521 (5)
Co2—N8	1.945 (3)	C9B—C10B	1.380 (6)
Co2—N7	1.946 (3)	C10B—C11B	1.379 (6)
Co2—N5	1.955 (3)	C10B—H10B	0.9500
Cl1—O11	1.432 (4)	C11B—C12B	1.397 (6)
Cl1—O9	1.434 (4)	C11B—H11B	0.9500
Cl1—O10	1.443 (4)	C12B—C13B	1.383 (6)
Cl1—O12	1.454 (4)	C12B—H12B	0.9500
O1A—C1A	1.314 (5)	C13B—C14B	1.516 (6)
O2A—C1A	1.218 (5)	C15—C16	1.375 (6)
O3A—C7A	1.226 (5)	C15—H15	0.9500
O4A—C7A	1.300 (5)	C16—C17	1.388 (6)
O5A—C8A	1.306 (5)	C16—H16	0.9500
O6A—C8A	1.215 (5)	C17—C18	1.380 (6)
O7A—C14A	1.223 (5)	C17—H17	0.9500
O8A—C14A	1.309 (5)	C18—C19	1.373 (6)
O1B—C1B	1.308 (5)	C18—H18	0.9500
O2B—C1B	1.223 (5)	C19—C20	1.467 (6)
O3B—C7B	1.232 (5)	C20—C21	1.382 (6)
O4B—C7B	1.306 (5)	C21—C22	1.377 (6)
O5B—C8B	1.309 (5)	C21—H21	0.9500
O6B—C8B	1.223 (5)	C22—C23	1.393 (6)
O7B—C14B	1.223 (5)	C22—H22	0.9500
O8B—C14B	1.310 (5)	C23—C24	1.382 (6)
O13—C47	1.124 (13)	C23—H23	0.9500
O14A—H14E	0.9378	C24—H24	0.9500
O14A—H14F	0.9441	C25—C26	1.390 (6)
O14B—H14G	0.8982	C25—H25	0.9500
O14B—H14H	0.9309	C26—C27	1.371 (7)
O15—H115	0.8500	C26—H26	0.9500
O15—H215	0.8500	C27—C28	1.392 (6)
N1A—C2A	1.330 (5)	C27—H27	0.9500
N1A—C6A	1.334 (5)	C28—C29	1.372 (6)
N2A—C13A	1.329 (5)	C28—H28	0.9500
N2A—C9A	1.332 (5)	C29—C30	1.476 (5)
N1B—C2B	1.319 (5)	C30—C31	1.384 (6)
N1B—C6B	1.328 (5)	C31—C32	1.388 (6)
N2B—C9B	1.328 (5)	C31—H31	0.9500
N2B—C13B	1.336 (5)	C32—C33	1.366 (6)
N3—C15	1.348 (5)	C32—H32	0.9500
N3—C19	1.354 (5)	C33—C34	1.380 (6)
N4—C24	1.352 (5)	C33—H33	0.9500
N4—C20	1.361 (5)	C34—H34	0.9500
N5—C25	1.353 (5)	C35—C36	1.386 (6)
N5—C29	1.356 (5)	C35—H35	0.9500
N6—C34	1.341 (5)	C36—C37	1.381 (6)
N6—C30	1.362 (5)	C36—H36	0.9500
N7—C35	1.344 (5)	C37—C38	1.383 (6)
N7—C39	1.360 (5)	C37—H37	0.9500
N8—C44	1.336 (5)	C38—C39	1.391 (6)
N8—C40	1.367 (5)	C38—H38	0.9500
N9—C47	1.357 (15)	C39—C40	1.462 (6)
N9—C45	1.508 (13)	C40—C41	1.382 (6)
N9—C46	1.510 (12)	C41—C42	1.376 (6)
C1A—C2A	1.512 (6)	C41—H41	0.9500
C2A—C3A	1.380 (5)	C42—C43	1.378 (6)
C3A—C4A	1.399 (6)	C42—H42	0.9500
C3A—H3A	0.9500	C43—C44	1.391 (6)
C4A—C5A	1.391 (6)	C43—H43	0.9500
C4A—H4A	0.9500	C44—H44	0.9500
C5A—C6A	1.378 (5)	C45—H45A	0.9800
C5A—H5A	0.9500	C45—H45B	0.9800
C6A—C7A	1.515 (6)	C45—H45C	0.9800
C8A—C9A	1.504 (6)	C46—H46A	0.9800
C9A—C10A	1.385 (5)	C46—H46B	0.9800
C10A—C11A	1.385 (6)	C46—H46C	0.9800
C10A—H10A	0.9500	C47—H47A	0.9500
			
N2A—Co1A—N1A	178.75 (15)	C3B—C2B—C1B	129.0 (4)
N2A—Co1A—O1A	95.68 (13)	C2B—C3B—C4B	117.5 (4)
N1A—Co1A—O1A	83.30 (13)	C2B—C3B—H3B	121.3
N2A—Co1A—O5A	83.46 (13)	C4B—C3B—H3B	121.3
N1A—Co1A—O5A	95.80 (13)	C5B—C4B—C3B	121.4 (4)
O1A—Co1A—O5A	89.85 (12)	C5B—C4B—H4B	119.3
N2A—Co1A—O8A	83.41 (13)	C3B—C4B—H4B	119.3
N1A—Co1A—O8A	97.34 (13)	C4B—C5B—C6B	117.6 (4)
O1A—Co1A—O8A	91.72 (12)	C4B—C5B—H5B	121.2
O5A—Co1A—O8A	166.86 (11)	C6B—C5B—H5B	121.2
N2A—Co1A—O4A	97.20 (13)	N1B—C6B—C5B	119.4 (4)
N1A—Co1A—O4A	83.82 (13)	N1B—C6B—C7B	111.2 (3)
O1A—Co1A—O4A	167.12 (12)	C5B—C6B—C7B	129.4 (4)
O5A—Co1A—O4A	91.49 (13)	O3B—C7B—O4B	124.6 (4)
O8A—Co1A—O4A	89.88 (12)	O3B—C7B—C6B	122.1 (4)
N1B—Co1B—N2B	174.28 (15)	O4B—C7B—C6B	113.3 (3)
N1B—Co1B—O8B	94.32 (13)	O6B—C8B—O5B	125.5 (4)
N2B—Co1B—O8B	83.66 (13)	O6B—C8B—C9B	121.6 (4)
N1B—Co1B—O4B	84.06 (13)	O5B—C8B—C9B	112.8 (3)
N2B—Co1B—O4B	90.60 (13)	N2B—C9B—C10B	119.4 (4)
O8B—Co1B—O4B	90.71 (12)	N2B—C9B—C8B	111.0 (3)
N1B—Co1B—O5B	98.43 (13)	C10B—C9B—C8B	129.4 (4)
N2B—Co1B—O5B	83.68 (13)	C11B—C10B—C9B	118.7 (4)
O8B—Co1B—O5B	167.26 (12)	C11B—C10B—H10B	120.6
O4B—Co1B—O5B	90.80 (12)	C9B—C10B—H10B	120.6
N1B—Co1B—O1B	82.94 (14)	C10B—C11B—C12B	120.8 (4)
N2B—Co1B—O1B	102.40 (13)	C10B—C11B—H11B	119.6
O8B—Co1B—O1B	90.57 (12)	C12B—C11B—H11B	119.6
O4B—Co1B—O1B	167.00 (12)	C13B—C12B—C11B	117.8 (4)
O5B—Co1B—O1B	90.80 (12)	C13B—C12B—H12B	121.1
N4—Co2—N3	83.20 (14)	C11B—C12B—H12B	121.1
N4—Co2—N6	92.47 (13)	N2B—C13B—C12B	119.6 (4)
N3—Co2—N6	89.89 (13)	N2B—C13B—C14B	110.3 (3)
N4—Co2—N8	94.83 (14)	C12B—C13B—C14B	129.9 (4)
N3—Co2—N8	175.94 (14)	O7B—C14B—O8B	124.4 (4)
N6—Co2—N8	93.75 (13)	O7B—C14B—C13B	122.6 (4)
N4—Co2—N7	89.64 (13)	O8B—C14B—C13B	113.0 (3)
N3—Co2—N7	93.27 (13)	N3—C15—C16	121.7 (4)
N6—Co2—N7	176.39 (14)	N3—C15—H15	119.2
N8—Co2—N7	83.15 (14)	C16—C15—H15	119.2
N4—Co2—N5	175.01 (13)	C15—C16—C17	119.5 (4)
N3—Co2—N5	94.97 (14)	C15—C16—H16	120.2
N6—Co2—N5	82.87 (14)	C17—C16—H16	120.2
N8—Co2—N5	87.28 (14)	C18—C17—C16	118.7 (4)
N7—Co2—N5	95.10 (14)	C18—C17—H17	120.6
O11—Cl1—O9	109.0 (2)	C16—C17—H17	120.6
O11—Cl1—O10	109.4 (2)	C19—C18—C17	119.3 (4)
O9—Cl1—O10	110.2 (2)	C19—C18—H18	120.4
O11—Cl1—O12	109.2 (3)	C17—C18—H18	120.4
O9—Cl1—O12	109.9 (2)	N3—C19—C18	122.1 (4)
O10—Cl1—O12	109.1 (2)	N3—C19—C20	113.9 (3)
C1A—O1A—Co1A	114.9 (3)	C18—C19—C20	124.0 (4)
C7A—O4A—Co1A	114.5 (2)	N4—C20—C21	121.7 (4)
C8A—O5A—Co1A	114.5 (3)	N4—C20—C19	113.9 (3)
C14A—O8A—Co1A	114.7 (2)	C21—C20—C19	124.4 (4)
C1B—O1B—Co1B	114.1 (3)	C22—C21—C20	119.5 (4)
C7B—O4B—Co1B	114.2 (3)	C22—C21—H21	120.2
C8B—O5B—Co1B	114.4 (2)	C20—C21—H21	120.2
C14B—O8B—Co1B	115.0 (2)	C21—C22—C23	119.2 (4)
H14E—O14A—H14F	113.6	C21—C22—H22	120.4
H14G—O14B—H14H	120.2	C23—C22—H22	120.4
H115—O15—H215	107.7	C24—C23—C22	119.0 (4)
C2A—N1A—C6A	124.3 (3)	C24—C23—H23	120.5
C2A—N1A—Co1A	118.3 (3)	C22—C23—H23	120.5
C6A—N1A—Co1A	117.4 (3)	N4—C24—C23	122.0 (4)
C13A—N2A—C9A	124.1 (3)	N4—C24—H24	119.0
C13A—N2A—Co1A	118.1 (3)	C23—C24—H24	119.0
C9A—N2A—Co1A	117.8 (3)	N5—C25—C26	121.5 (4)
C2B—N1B—C6B	124.0 (3)	N5—C25—H25	119.2
C2B—N1B—Co1B	118.7 (3)	C26—C25—H25	119.2
C6B—N1B—Co1B	117.1 (3)	C27—C26—C25	120.1 (4)
C9B—N2B—C13B	123.6 (3)	C27—C26—H26	120.0
C9B—N2B—Co1B	117.3 (3)	C25—C26—H26	120.0
C13B—N2B—Co1B	117.5 (3)	C26—C27—C28	118.4 (4)
C15—N3—C19	118.6 (3)	C26—C27—H27	120.8
C15—N3—Co2	126.9 (3)	C28—C27—H27	120.8
C19—N3—Co2	114.4 (3)	C29—C28—C27	119.5 (4)
C24—N4—C20	118.6 (3)	C29—C28—H28	120.2
C24—N4—Co2	126.9 (3)	C27—C28—H28	120.2
C20—N4—Co2	114.3 (3)	N5—C29—C28	122.3 (4)
C25—N5—C29	118.1 (4)	N5—C29—C30	113.9 (4)
C25—N5—Co2	127.3 (3)	C28—C29—C30	123.8 (4)
C29—N5—Co2	114.6 (2)	N6—C30—C31	122.1 (4)
C34—N6—C30	117.9 (3)	N6—C30—C29	114.0 (3)
C34—N6—Co2	127.4 (3)	C31—C30—C29	123.9 (4)
C30—N6—Co2	114.6 (2)	C30—C31—C32	118.7 (4)
C35—N7—C39	119.3 (3)	C30—C31—H31	120.7
C35—N7—Co2	126.8 (3)	C32—C31—H31	120.7
C39—N7—Co2	114.0 (3)	C33—C32—C31	119.2 (4)
C44—N8—C40	118.2 (3)	C33—C32—H32	120.4
C44—N8—Co2	127.5 (3)	C31—C32—H32	120.4
C40—N8—Co2	114.3 (3)	C32—C33—C34	119.6 (4)
C47—N9—C45	120.5 (9)	C32—C33—H33	120.2
C47—N9—C46	117.1 (10)	C34—C33—H33	120.2
C45—N9—C46	121.5 (9)	N6—C34—C33	122.4 (4)
O2A—C1A—O1A	124.1 (4)	N6—C34—H34	118.8
O2A—C1A—C2A	122.9 (4)	C33—C34—H34	118.8
O1A—C1A—C2A	112.9 (3)	N7—C35—C36	121.4 (4)
N1A—C2A—C3A	119.6 (4)	N7—C35—H35	119.3
N1A—C2A—C1A	110.5 (3)	C36—C35—H35	119.3
C3A—C2A—C1A	129.8 (4)	C37—C36—C35	119.7 (4)
C2A—C3A—C4A	117.4 (4)	C37—C36—H36	120.2
C2A—C3A—H3A	121.3	C35—C36—H36	120.2
C4A—C3A—H3A	121.3	C36—C37—C38	119.1 (4)
C5A—C4A—C3A	121.3 (4)	C36—C37—H37	120.5
C5A—C4A—H4A	119.3	C38—C37—H37	120.5
C3A—C4A—H4A	119.3	C37—C38—C39	119.2 (4)
C6A—C5A—C4A	118.0 (4)	C37—C38—H38	120.4
C6A—C5A—H5A	121.0	C39—C38—H38	120.4
C4A—C5A—H5A	121.0	N7—C39—C38	121.3 (4)
N1A—C6A—C5A	119.3 (4)	N7—C39—C40	114.8 (3)
N1A—C6A—C7A	110.7 (3)	C38—C39—C40	124.0 (4)
C5A—C6A—C7A	129.9 (4)	N8—C40—C41	121.7 (4)
O3A—C7A—O4A	125.5 (4)	N8—C40—C39	113.7 (3)
O3A—C7A—C6A	121.0 (4)	C41—C40—C39	124.5 (4)
O4A—C7A—C6A	113.5 (3)	C42—C41—C40	119.6 (4)
O6A—C8A—O5A	124.5 (4)	C42—C41—H41	120.2
O6A—C8A—C9A	122.1 (4)	C40—C41—H41	120.2
O5A—C8A—C9A	113.5 (3)	C41—C42—C43	118.8 (4)
N2A—C9A—C10A	119.4 (4)	C41—C42—H42	120.6
N2A—C9A—C8A	110.7 (3)	C43—C42—H42	120.6
C10A—C9A—C8A	129.9 (4)	C42—C43—C44	119.4 (4)
C9A—C10A—C11A	118.3 (4)	C42—C43—H43	120.3
C9A—C10A—H10A	120.9	C44—C43—H43	120.3
C11A—C10A—H10A	120.9	N8—C44—C43	122.2 (4)
C10A—C11A—C12A	121.0 (4)	N8—C44—H44	118.9
C10A—C11A—H11A	119.5	C43—C44—H44	118.9
C12A—C11A—H11A	119.5	N9—C45—H45A	109.5
C13A—C12A—C11A	117.5 (4)	N9—C45—H45B	109.5
C13A—C12A—H12A	121.2	H45A—C45—H45B	109.5
C11A—C12A—H12A	121.2	N9—C45—H45C	109.5
N2A—C13A—C12A	119.7 (4)	H45A—C45—H45C	109.5
N2A—C13A—C14A	110.6 (3)	H45B—C45—H45C	109.5
C12A—C13A—C14A	129.7 (4)	N9—C46—H46A	109.5
O7A—C14A—O8A	124.7 (4)	N9—C46—H46B	109.5
O7A—C14A—C13A	122.2 (4)	H46A—C46—H46B	109.5
O8A—C14A—C13A	113.1 (3)	N9—C46—H46C	109.5
O2B—C1B—O1B	125.1 (4)	H46A—C46—H46C	109.5
O2B—C1B—C2B	121.5 (4)	H46B—C46—H46C	109.5
O1B—C1B—C2B	113.4 (4)	O13—C47—N9	124.2 (13)
N1B—C2B—C3B	120.1 (4)	O13—C47—H47A	117.9
N1B—C2B—C1B	110.8 (3)	N9—C47—H47A	117.9
			
N2A—Co1A—O1A—C1A	−176.2 (3)	O6A—C8A—C9A—N2A	176.8 (4)
N1A—Co1A—O1A—C1A	3.1 (3)	O5A—C8A—C9A—N2A	−1.8 (5)
O5A—Co1A—O1A—C1A	−92.8 (3)	O6A—C8A—C9A—C10A	−2.8 (7)
O8A—Co1A—O1A—C1A	100.3 (3)	O5A—C8A—C9A—C10A	178.6 (4)
O4A—Co1A—O1A—C1A	3.3 (7)	N2A—C9A—C10A—C11A	−0.1 (6)
N2A—Co1A—O4A—C7A	179.6 (3)	C8A—C9A—C10A—C11A	179.5 (4)
N1A—Co1A—O4A—C7A	0.4 (3)	C9A—C10A—C11A—C12A	−0.6 (6)
O1A—Co1A—O4A—C7A	0.1 (7)	C10A—C11A—C12A—C13A	1.1 (6)
O5A—Co1A—O4A—C7A	96.0 (3)	C9A—N2A—C13A—C12A	0.3 (6)
O8A—Co1A—O4A—C7A	−97.0 (3)	Co1A—N2A—C13A—C12A	178.0 (3)
N2A—Co1A—O5A—C8A	1.3 (3)	C9A—N2A—C13A—C14A	−178.5 (3)
N1A—Co1A—O5A—C8A	−177.7 (3)	Co1A—N2A—C13A—C14A	−0.8 (4)
O1A—Co1A—O5A—C8A	−94.4 (3)	C11A—C12A—C13A—N2A	−0.9 (6)
O8A—Co1A—O5A—C8A	2.5 (7)	C11A—C12A—C13A—C14A	177.6 (4)
O4A—Co1A—O5A—C8A	98.4 (3)	Co1A—O8A—C14A—O7A	179.8 (3)
N2A—Co1A—O8A—C14A	1.0 (3)	Co1A—O8A—C14A—C13A	−1.6 (4)
N1A—Co1A—O8A—C14A	179.9 (3)	N2A—C13A—C14A—O7A	−179.8 (4)
O1A—Co1A—O8A—C14A	96.5 (3)	C12A—C13A—C14A—O7A	1.6 (7)
O5A—Co1A—O8A—C14A	−0.3 (7)	N2A—C13A—C14A—O8A	1.6 (5)
O4A—Co1A—O8A—C14A	−96.3 (3)	C12A—C13A—C14A—O8A	−177.1 (4)
N1B—Co1B—O1B—C1B	1.8 (3)	Co1B—O1B—C1B—O2B	179.8 (3)
N2B—Co1B—O1B—C1B	−176.1 (3)	Co1B—O1B—C1B—C2B	−1.2 (4)
O8B—Co1B—O1B—C1B	−92.4 (3)	C6B—N1B—C2B—C3B	−1.4 (6)
O4B—Co1B—O1B—C1B	3.2 (7)	Co1B—N1B—C2B—C3B	−176.7 (3)
O5B—Co1B—O1B—C1B	100.2 (3)	C6B—N1B—C2B—C1B	177.3 (3)
N1B—Co1B—O4B—C7B	−1.6 (3)	Co1B—N1B—C2B—C1B	2.0 (4)
N2B—Co1B—O4B—C7B	176.3 (3)	O2B—C1B—C2B—N1B	178.6 (4)
O8B—Co1B—O4B—C7B	92.7 (3)	O1B—C1B—C2B—N1B	−0.4 (5)
O5B—Co1B—O4B—C7B	−100.0 (3)	O2B—C1B—C2B—C3B	−2.9 (6)
O1B—Co1B—O4B—C7B	−2.9 (7)	O1B—C1B—C2B—C3B	178.2 (4)
N1B—Co1B—O5B—C8B	−169.6 (3)	N1B—C2B—C3B—C4B	0.8 (6)
N2B—Co1B—O5B—C8B	5.0 (3)	C1B—C2B—C3B—C4B	−177.6 (4)
O8B—Co1B—O5B—C8B	11.3 (7)	C2B—C3B—C4B—C5B	0.5 (6)
O4B—Co1B—O5B—C8B	−85.5 (3)	C3B—C4B—C5B—C6B	−1.3 (6)
O1B—Co1B—O5B—C8B	107.4 (3)	C2B—N1B—C6B—C5B	0.6 (6)
N1B—Co1B—O8B—C14B	172.2 (3)	Co1B—N1B—C6B—C5B	176.0 (3)
N2B—Co1B—O8B—C14B	−2.5 (3)	C2B—N1B—C6B—C7B	−178.1 (3)
O4B—Co1B—O8B—C14B	88.1 (3)	Co1B—N1B—C6B—C7B	−2.8 (4)
O5B—Co1B—O8B—C14B	−8.7 (7)	C4B—C5B—C6B—N1B	0.7 (6)
O1B—Co1B—O8B—C14B	−104.9 (3)	C4B—C5B—C6B—C7B	179.2 (4)
O1A—Co1A—N1A—C2A	−1.9 (3)	Co1B—O4B—C7B—O3B	−179.0 (3)
O5A—Co1A—N1A—C2A	87.3 (3)	Co1B—O4B—C7B—C6B	0.5 (4)
O8A—Co1A—N1A—C2A	−92.7 (3)	N1B—C6B—C7B—O3B	−179.1 (4)
O4A—Co1A—N1A—C2A	178.2 (3)	C5B—C6B—C7B—O3B	2.3 (6)
O1A—Co1A—N1A—C6A	179.5 (3)	N1B—C6B—C7B—O4B	1.4 (5)
O5A—Co1A—N1A—C6A	−91.3 (3)	C5B—C6B—C7B—O4B	−177.2 (4)
O8A—Co1A—N1A—C6A	88.6 (3)	Co1B—O5B—C8B—O6B	177.5 (4)
O4A—Co1A—N1A—C6A	−0.4 (3)	Co1B—O5B—C8B—C9B	−1.0 (4)
O1A—Co1A—N2A—C13A	−91.1 (3)	C13B—N2B—C9B—C10B	−0.4 (6)
O5A—Co1A—N2A—C13A	179.7 (3)	Co1B—N2B—C9B—C10B	−165.3 (3)
O8A—Co1A—N2A—C13A	0.0 (3)	C13B—N2B—C9B—C8B	174.8 (3)
O4A—Co1A—N2A—C13A	89.1 (3)	Co1B—N2B—C9B—C8B	9.9 (4)
O1A—Co1A—N2A—C9A	86.7 (3)	O6B—C8B—C9B—N2B	175.9 (4)
O5A—Co1A—N2A—C9A	−2.5 (3)	O5B—C8B—C9B—N2B	−5.5 (5)
O8A—Co1A—N2A—C9A	177.8 (3)	O6B—C8B—C9B—C10B	−9.4 (7)
O4A—Co1A—N2A—C9A	−93.1 (3)	O5B—C8B—C9B—C10B	169.2 (4)
O8B—Co1B—N1B—C2B	87.9 (3)	N2B—C9B—C10B—C11B	−1.0 (6)
O4B—Co1B—N1B—C2B	178.1 (3)	C8B—C9B—C10B—C11B	−175.2 (4)
O5B—Co1B—N1B—C2B	−92.0 (3)	C9B—C10B—C11B—C12B	0.7 (6)
O1B—Co1B—N1B—C2B	−2.2 (3)	C10B—C11B—C12B—C13B	0.8 (6)
O8B—Co1B—N1B—C6B	−87.7 (3)	C9B—N2B—C13B—C12B	2.0 (6)
O4B—Co1B—N1B—C6B	2.5 (3)	Co1B—N2B—C13B—C12B	166.9 (3)
O5B—Co1B—N1B—C6B	92.5 (3)	C9B—N2B—C13B—C14B	−174.0 (3)
O1B—Co1B—N1B—C6B	−177.8 (3)	Co1B—N2B—C13B—C14B	−9.1 (4)
O8B—Co1B—N2B—C9B	172.7 (3)	C11B—C12B—C13B—N2B	−2.1 (6)
O4B—Co1B—N2B—C9B	82.1 (3)	C11B—C12B—C13B—C14B	173.0 (4)
O5B—Co1B—N2B—C9B	−8.7 (3)	Co1B—O8B—C14B—O7B	179.2 (3)
O1B—Co1B—N2B—C9B	−98.1 (3)	Co1B—O8B—C14B—C13B	−1.8 (4)
O8B—Co1B—N2B—C13B	6.9 (3)	N2B—C13B—C14B—O7B	−174.1 (4)
O4B—Co1B—N2B—C13B	−83.8 (3)	C12B—C13B—C14B—O7B	10.4 (7)
O5B—Co1B—N2B—C13B	−174.5 (3)	N2B—C13B—C14B—O8B	6.8 (5)
O1B—Co1B—N2B—C13B	96.1 (3)	C12B—C13B—C14B—O8B	−168.7 (4)
N4—Co2—N3—C15	176.3 (3)	C19—N3—C15—C16	0.9 (6)
N6—Co2—N3—C15	−91.2 (3)	Co2—N3—C15—C16	−177.2 (3)
N7—Co2—N3—C15	87.0 (3)	N3—C15—C16—C17	−0.3 (6)
N5—Co2—N3—C15	−8.4 (3)	C15—C16—C17—C18	−0.3 (7)
N4—Co2—N3—C19	−1.9 (3)	C16—C17—C18—C19	0.3 (7)
N6—Co2—N3—C19	90.6 (3)	C15—N3—C19—C18	−0.9 (6)
N7—Co2—N3—C19	−91.2 (3)	Co2—N3—C19—C18	177.5 (3)
N5—Co2—N3—C19	173.4 (3)	C15—N3—C19—C20	−179.2 (3)
N3—Co2—N4—C24	179.8 (3)	Co2—N3—C19—C20	−0.8 (4)
N6—Co2—N4—C24	90.2 (3)	C17—C18—C19—N3	0.3 (7)
N8—Co2—N4—C24	−3.8 (3)	C17—C18—C19—C20	178.5 (4)
N7—Co2—N4—C24	−86.9 (3)	C24—N4—C20—C21	−2.3 (6)
N3—Co2—N4—C20	4.5 (3)	Co2—N4—C20—C21	173.4 (3)
N6—Co2—N4—C20	−85.1 (3)	C24—N4—C20—C19	178.2 (3)
N8—Co2—N4—C20	−179.1 (3)	Co2—N4—C20—C19	−6.1 (4)
N7—Co2—N4—C20	97.8 (3)	N3—C19—C20—N4	4.5 (5)
N3—Co2—N5—C25	91.6 (3)	C18—C19—C20—N4	−173.7 (4)
N6—Co2—N5—C25	−179.2 (3)	N3—C19—C20—C21	−175.0 (4)
N8—Co2—N5—C25	−85.0 (3)	C18—C19—C20—C21	6.8 (7)
N7—Co2—N5—C25	−2.2 (3)	N4—C20—C21—C22	1.6 (7)
N3—Co2—N5—C29	−90.5 (3)	C19—C20—C21—C22	−178.9 (4)
N6—Co2—N5—C29	−1.3 (3)	C20—C21—C22—C23	0.3 (7)
N8—Co2—N5—C29	92.9 (3)	C21—C22—C23—C24	−1.4 (7)
N7—Co2—N5—C29	175.7 (3)	C20—N4—C24—C23	1.1 (6)
N4—Co2—N6—C34	−0.8 (3)	Co2—N4—C24—C23	−174.0 (3)
N3—Co2—N6—C34	−84.0 (3)	C22—C23—C24—N4	0.8 (6)
N8—Co2—N6—C34	94.2 (3)	C29—N5—C25—C26	1.8 (6)
N5—Co2—N6—C34	−179.0 (3)	Co2—N5—C25—C26	179.7 (3)
N4—Co2—N6—C30	−179.0 (3)	N5—C25—C26—C27	0.6 (6)
N3—Co2—N6—C30	97.8 (3)	C25—C26—C27—C28	−2.1 (7)
N8—Co2—N6—C30	−84.0 (3)	C26—C27—C28—C29	1.2 (6)
N5—Co2—N6—C30	2.8 (3)	C25—N5—C29—C28	−2.8 (6)
N4—Co2—N7—C35	−82.4 (3)	Co2—N5—C29—C28	179.1 (3)
N3—Co2—N7—C35	0.7 (3)	C25—N5—C29—C30	177.7 (3)
N8—Co2—N7—C35	−177.3 (4)	Co2—N5—C29—C30	−0.4 (4)
N5—Co2—N7—C35	96.0 (3)	C27—C28—C29—N5	1.4 (6)
N4—Co2—N7—C39	97.9 (3)	C27—C28—C29—C30	−179.2 (4)
N3—Co2—N7—C39	−179.0 (3)	C34—N6—C30—C31	−2.7 (6)
N8—Co2—N7—C39	2.9 (3)	Co2—N6—C30—C31	175.7 (3)
N5—Co2—N7—C39	−83.7 (3)	C34—N6—C30—C29	177.9 (3)
N4—Co2—N8—C44	91.8 (4)	Co2—N6—C30—C29	−3.7 (4)
N6—Co2—N8—C44	−1.0 (4)	N5—C29—C30—N6	2.7 (5)
N7—Co2—N8—C44	−179.1 (4)	C28—C29—C30—N6	−176.7 (4)
N5—Co2—N8—C44	−83.7 (4)	N5—C29—C30—C31	−176.7 (4)
N4—Co2—N8—C40	−90.7 (3)	C28—C29—C30—C31	3.9 (6)
N6—Co2—N8—C40	176.5 (3)	N6—C30—C31—C32	0.8 (6)
N7—Co2—N8—C40	−1.6 (3)	C29—C30—C31—C32	−179.9 (4)
N5—Co2—N8—C40	93.9 (3)	C30—C31—C32—C33	1.4 (6)
Co1A—O1A—C1A—O2A	175.7 (3)	C31—C32—C33—C34	−1.6 (6)
Co1A—O1A—C1A—C2A	−3.6 (4)	C30—N6—C34—C33	2.6 (6)
C6A—N1A—C2A—C3A	−0.3 (6)	Co2—N6—C34—C33	−175.6 (3)
Co1A—N1A—C2A—C3A	−178.9 (3)	C32—C33—C34—N6	−0.4 (6)
C6A—N1A—C2A—C1A	178.9 (3)	C39—N7—C35—C36	3.3 (6)
Co1A—N1A—C2A—C1A	0.4 (4)	Co2—N7—C35—C36	−176.4 (3)
O2A—C1A—C2A—N1A	−177.2 (4)	N7—C35—C36—C37	0.1 (7)
O1A—C1A—C2A—N1A	2.1 (4)	C35—C36—C37—C38	−2.4 (7)
O2A—C1A—C2A—C3A	2.0 (6)	C36—C37—C38—C39	1.5 (8)
O1A—C1A—C2A—C3A	−178.7 (4)	C35—N7—C39—C38	−4.2 (6)
N1A—C2A—C3A—C4A	−0.4 (5)	Co2—N7—C39—C38	175.5 (4)
C1A—C2A—C3A—C4A	−179.5 (4)	C35—N7—C39—C40	176.6 (4)
C2A—C3A—C4A—C5A	0.6 (6)	Co2—N7—C39—C40	−3.7 (5)
C3A—C4A—C5A—C6A	−0.1 (6)	C37—C38—C39—N7	1.9 (7)
C2A—N1A—C6A—C5A	0.8 (6)	C37—C38—C39—C40	−179.0 (5)
Co1A—N1A—C6A—C5A	179.3 (3)	C44—N8—C40—C41	−2.0 (6)
C2A—N1A—C6A—C7A	−178.1 (3)	Co2—N8—C40—C41	−179.8 (4)
Co1A—N1A—C6A—C7A	0.4 (4)	C44—N8—C40—C39	177.8 (4)
C4A—C5A—C6A—N1A	−0.5 (6)	Co2—N8—C40—C39	0.1 (5)
C4A—C5A—C6A—C7A	178.2 (4)	N7—C39—C40—N8	2.4 (6)
Co1A—O4A—C7A—O3A	−179.6 (3)	C38—C39—C40—N8	−176.8 (4)
Co1A—O4A—C7A—C6A	−0.2 (4)	N7—C39—C40—C41	−177.8 (4)
N1A—C6A—C7A—O3A	179.3 (4)	C38—C39—C40—C41	3.1 (8)
C5A—C6A—C7A—O3A	0.5 (7)	N8—C40—C41—C42	2.2 (7)
N1A—C6A—C7A—O4A	−0.1 (5)	C39—C40—C41—C42	−177.6 (5)
C5A—C6A—C7A—O4A	−178.9 (4)	C40—C41—C42—C43	−0.4 (8)
Co1A—O5A—C8A—O6A	−178.6 (4)	C41—C42—C43—C44	−1.6 (7)
Co1A—O5A—C8A—C9A	−0.1 (4)	C40—N8—C44—C43	0.0 (6)
C13A—N2A—C9A—C10A	0.2 (6)	Co2—N8—C44—C43	177.4 (3)
Co1A—N2A—C9A—C10A	−177.4 (3)	C42—C43—C44—N8	1.8 (7)
C13A—N2A—C9A—C8A	−179.4 (3)	C45—N9—C47—O13	9 (2)
Co1A—N2A—C9A—C8A	2.9 (4)		

### Hydrogen-bond geometry (Å, º)

**Table:**

*D*—H···*A*	*D*—H	H···*A*	*D*···*A*	*D*—H···*A*
O14*A*—H14*E*···O12	0.94	2.11	3.017 (9)	164
O14*A*—H14*F*···O3*A*^i^	0.94	1.97	2.861 (9)	156
O15—H115···O7*A*^ii^	0.85	2.20	3.029 (13)	164
O15—H215···O2*B*	0.85	2.07	2.842 (13)	152

Symmetry codes: (i) *x*, *y*+1, *z*; (ii) −*x*, −*y*, −*z*+1.
